# Functional Characterization, Antimicrobial Effects, and Potential Antibacterial Mechanisms of *Np*HM4, a Derived Peptide of *Nautilus pompilius* Hemocyanin

**DOI:** 10.3390/md20070459

**Published:** 2022-07-16

**Authors:** Chun Yuan, Xiaoying Zheng, Kunna Liu, Wenbin Yuan, Yang Zhang, Fan Mao, Yongbo Bao

**Affiliations:** 1 Zhejiang Key Laboratory of Aquatic Germplasm Resources, College of Biological and Environmental Sciences, Zhejiang Wanli University, Ningbo 315100, China; 2020881038@zwu.edu.cn (C.Y.); xyzheng20@163.com (X.Z.); yuanwenbin2021@163.com (W.Y.); 2 Ninghai Institute of Mariculture Breeding and Seed Industry, Zhejiang Wanli University, Ningbo 315604, China; 3 School of Marine Science, Ningbo University, Ningbo 315211, China; 4 CAS Key Laboratory of Tropical Marine Bio-Resources and Ecology and Guangdong Provincial Key Laboratory of Applied Marine Biology, South China Sea Institute of Oceanology, Innovation Academy of South China Sea Ecology and Environmental Engineering, Chinese Academy of Sciences, Guangzhou 510301, China; liukunna18@mails.ucas.ac.cn (K.L.); yzhang@scsio.ac.cn (Y.Z.); 5 Southern Marine Science and Engineering Guangdong Laboratory (Guangzhou), Guangzhou 510301, China

**Keywords:** *Nautilus pompilius*, hemocyanin, *Np*HM4, antimicrobial peptides, cytotoxicity

## Abstract

Hemocyanins present in the hemolymph of invertebrates are multifunctional proteins that are responsible for oxygen transport and play crucial roles in the immune system. They have also been identified as a source of antimicrobial peptides during infection in mollusks. Hemocyanin has also been identified in the cephalopod ancestor Nautilus, but antimicrobial peptides derived from the hemocyanin of *Nautilus pompilius* have not been reported. Here, the bactericidal activity of six predicted peptides from *N. pompilius* hemocyanin and seven mutant peptides was analyzed. Among those peptides, a mutant peptide with 15 amino acids (1RVFAGFLRHGIKRSR15), *Np*HM4, showed relatively high antibacterial activity. *Np*HM4 was determined to have typical antimicrobial peptide characteristics, including a positive charge (+5.25) and a high hydrophobic residue ratio (40%), and it was predicted to form an alpha-helical structure. In addition, *Np*HM4 exhibited significant antibacterial activity against Gram-negative bacteria (MBC = 30 μM for *Vibrio alginolyticus*), with no cytotoxicity to mammalian cells even at a high concentration of 180 µM. Upon contact with *V. alginolyticus* cells, we confirmed that the bactericidal activity of *Np*HM4 was coupled with membrane permeabilization, which was further confirmed via ultrastructural images using a scanning electron microscope. Therefore, our study provides a rationalization for the development and optimization of antimicrobial peptide from the cephalopod ancestor Nautilus, paving the way for future novel AMP development with broad applications.

## 1. Introduction

The increasing emergence and dissemination of antibiotic resistance of microorganisms has become a serious threat to global public health [1,2]. Thus, there is an urgent need for the development of new antimicrobial agents to overcome this problem. Antimicrobial peptides (AMPs) are widely distributed small-molecule peptides that are produced as a first line of defense by all multi-cellular organisms [3]. With their specific properties of rapid action, broad-spectrum antimicrobial activity, and infrequent development of resistance [4,5], AMP-based drugs are obvious templates for a wide range of antimicrobial agents and biomedical applications. Natural AMPs possess similar sequences and structural characteristics, with lengths between 12 and 50 amino acids; generally containing cationic and hydrophobic residues [6]. Most antimicrobial peptides are amphiphilic, with hydrophilic as well as hydrophobic surfaces, displaying variations in structure and size. Due to their amphiphilic structure, these peptides normally operate by forming pores in microbial membranes or otherwise disrupting membrane integrity [7].

Hemocyanins are large, copper-containing, multi-subunit oxygen carrier proteins, found in some mollusks and arthropods; which also play essential roles in the immune system [8]. Previous studies have proved that the immune activity of marine invertebrates’ hemocyanin ground on a variety of mechanisms, including glycosylation modification [9], strong phagocytic capacity [10], and enzymatic induction, that cause the generation of reactive oxygen species [11]. Recently, AMPs have been found to be derived from hemocyanin, which are constitutively synthesized in hemocytes and stored in granules [12]. Hemocyanin-derived AMPs have been studied in several invertebrates, such as the haliotisin peptide from the hemocyanin of abalones, which harbors bactericidal potential [13]. The Hmc364-382 peptide, derived from the hemocyanin of the shrimp *Penaeus monodon*, also showed promising activity against *Trypanosoma cruzi* [12].

All cephalopods are highly sensitive to diseases and toxins in water, because they possess a nonadaptive immune system [14,15]. The immune system of cephalopods works based on cellular factors [16]. In addition, dissolved molecules in the serum (opsonins, agglutinins, and lysozyme) also contribute to the immune response of cephalopods [17]. In cephalopods, different AMPs have been described, such as the GR21 peptide from cuttlefish [18] and the Octominin peptide derived from octopus [19]. Nautili are the oldest known living cephalopods [20]. Among them, *Nautilus pompilius* is the most widely distributed species [21]. However, no AMP has been identified in Nautili yet. Recently, Yang et al. [22] sequenced and analyzed the genome-wide of *N. pompilius*, revealing a highly complex and comprehensive innate immune system. The hemocyanin of Nautili has been reported as a cyclic decolymer of 350 kDa polypeptide subunits, consisting of seven disparate functional units (FU-a to FU-g) [23]. Thus, the development of AMPs in Nautili is a promising and attractive area of research. Furthermore, the modification of native peptides or AMP sequences, such as amino acid substitutions [24], can be performed to develop ideal AMPs [25].

Here, we utilized bioinformatic tools to predict AMPs from the hemocyanin of *N. pompilius*. Forty-four potential peptides were identified. Among them, peptide *Np*HN5 was singled out with relatively significant antibacterial activity. Then, we designed a series of mutated peptides using *Np*HN5 as a scaffold, aiming to improve the effectiveness and safety of hemocyanin-derived AMPs from *N. pompilius*. We also explored the bactericidal mechanism of the most promising AMP, *Np*HM4, as well as its cytotoxicity to mammalian cells. Thus, in our study, we developed AMPs from the hemocyanin of *N. pompilius* and provided evidence supporting their potential antibacterial application.

## 2. Results

### 2.1. In Silico Predicted Peptides with Antimicrobial Activity

In our study, two online software packages (the antibacterial peptides server, AntiBP, and the collection of anti-microbial peptides server, CAMP) were applied for the prediction of antibacterial peptides. The AntiBP server was used to search for putative AMP fragments among the large subunit sequence of the hemocyanin of *N. pompilius*, while the CAMP server was applied to evaluate the reliability of the predicted active polypeptide fragments. Forty-four potential peptides (N1–N44), ranging from 1.5 to 2.0 kDa, were predicted from the hemocyanin of *N. pompilius* using the AntiBP server, and all of the predicted peptides were ranked from high to low according to the probability score of AMP, predicted using the CAMP server (Table S1). The higher the score is, the greater the possibility of the peptide being antimicrobial. Among them, N1, N2, N3, N4, N6, and N8 were recombined, because of their high similarity in sequence, to form a new peptide sequence, VFAGFMLHGFKKSALV, which was named *Np*HN1. Likewise, N10 and N21 were recombined as FASFRLSGIHTSANVKVL, which was named *Np*HN2. Then, the antibacterial potential of *Np*HN1 and *Np*HN2 was further validated, and the result (Table S2) showed that the predicted score of *Np*HN1 was the same as N1 but higher than N2, N3, N4, N6, and N8. Additionally, *Np*HN2 had the highest AMP score, compared with N10 and N21.

The APD3 software was then used to calculate the hydrophobic ratio, the net charge, the molecular weight, and the other parameters for peptides N1–N44. According to the APD3 prediction (Table S1), the molecular weights of all the sequences ranged from 1586.85 (N14) to 1977.30 (N43), the total hydrophobic ratios ranged between 33% and 60%, and the total net charges ranged from +1 (N36) to +4.25 (N44). Given the fact that huge amounts of known AMPs have secondary conformational structures, such as α-helix structures [26,27] or β-chain structures [28,29], AMPs derived from the hemocyanin of *N. pompilius* were further screened using the secondary conformational structures obtained from APD3 (Tables S1 and S2). The results showed that *Np*HN1 and *Np*HN2 may form α-helix structures. Moreover, among the antimicrobial peptides with high CAMP scores (score > 0.8), N5, N7, N9, N11, and N14 may form α-helix structures or β-chain structures, suggesting their predominant potential as antimicrobial peptides, while N12, N13, and N15 do not form α-helix or β-chain structures. We ultimately selected six peptides, N5, N7, N9, N14, *Np*HN1, and *Np*HN2, for synthesis and antibacterial screening. For the sake of unified naming, N5, N7, N9, and N14 were renamed *Np*HN3, *Np*HN4, *Np*HN5, and *Np*HN6, respectively. The peptide characteristics of *Np*HN1–*Np*HN6 are shown in Table S2. Among the six predicted antimicrobial peptides, *Np*HN5 had the highest net charge of +3.25 with a 53% proportion of hydrophobic amino acids.

### 2.2. Antibacterial Activity of the Six Predicted Antimicrobial Peptides (NpHN1–NpHN6)

In order to explore the antibacterial activity of the six predicted AMPs, the minimal inhibitory concentrations (MICs) and the minimal bactericidal concentrations (MBCs) of chemically synthesized *Np*HN1–*Np*HN6 peptides were measured against Gram-negative bacteria (*Vibrio alginolyticus* and *Escherichia coli*). The results showed that only *Np*HN5 had certain antibacterial activity against the tested bacteria (Table 1), with an MBC of 250 μM against *V. alginolyticus*. However, the MBC of *Np*HN5 was not comparable to that of GR21 (GSTSFHLIYNKWFAVKRRRKR), a known AMP derived from cuttlefish [18]. The GR21 peptide showed a relatively low MBC (below 25 μM) on several strains, including *E. coli*, *Bacillus megaterium*, and *Staphylococcus aureus*. We noted that GR21 was the least hydrophobic and the most cationic peptide of the nine peptides validated by Baptiste et al. [18], indicating that hydrophobicity and positive charge play important roles in antibacterial activity. Thus, we decided to modify the sequence of *Np*HN5 to improve its antibacterial activity, aiming to develop novel and effective antimicrobial peptides from the hemocyanin of *N. pompilius*.

### 2.3. Sequence Analysis of Mutant AMPs Derived from NpHN5

**Figure 1:**
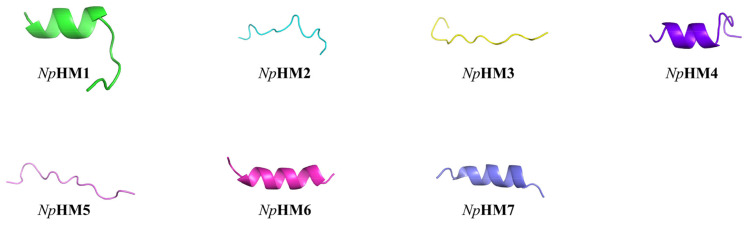
Three-dimensional structure simulation of seven mutant peptides (*Np*HM1, *Np*HM2, *Np*HM3, *Np*HM4, *Np*HM5, *Np*HM6, and *Np*HM7), predicted using I-TASSER server.

In order to improve the bactericidal activity of *Np*HN5, a series of new candidate AMP (*Np*HM1–*Np*HM7) sequences were obtained via amino acid substitutions on the basis of the *Np*HN5 sequence (Table 2). Within the series, *Np*HM1, *Np*HM4, *Np*HM6, and *Np*HM7 were able to form α-helices according to the I-TASSER prediction (Table 2, Figure 1). However, *Np*HM2, *Np*HM3, and *Np*HM5 all formed β-chain structures (Table 2, Figure 1). *Np*HM4 displayed the highest mean hydrophobic moment (Table 2), formed a helical structure in the 3D structural projection, and had a cationic polar face (Figure S1). In contrast, *Np*HM5 showed the lowest average hydrophobic moment of 0.160. *Np*HM6 displayed an increase in positive charge from +3.25 (*Np*HN5) to +5.25. *Np*HM7, obtained via the substitution of three amino acids (Phe^3^, Leu^8^, and Ala^15^) with arginines, possessed the highest positive charge (+6.25) and the lowest hydrophobicity (33%).

### 2.4. Bactericidal Activity of Seven Mutant AMPs

**Figure 2:**
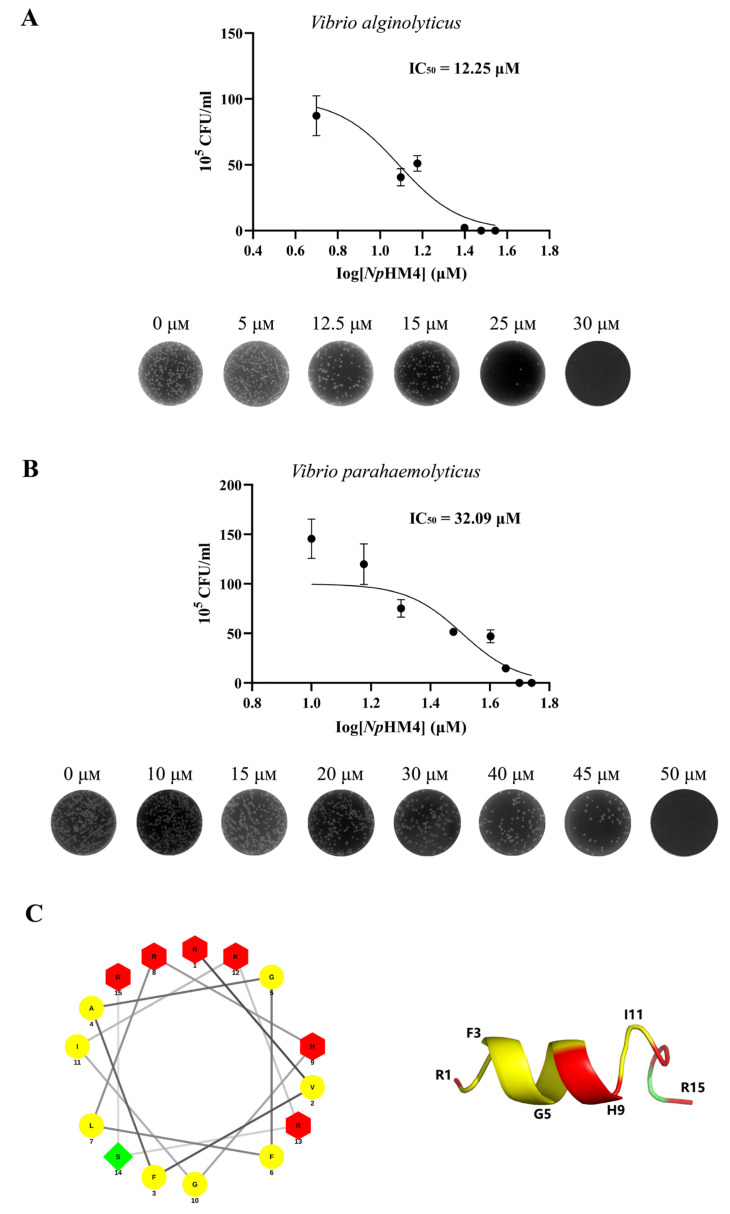
The antibacterial activity and predicted structure of peptide *Np*HM4. IC_50_ (the half maximal inhibitory concentration) values are indicated, which represent the concentration of *Np*HM4 that is required for 50% inhibition of the microbial samples. (**A**) with *Vibrio alginolyticus*; (**B**) with *Vibrio* *parahaemolyticus*. The experiments were carried out in triplicate, repeated at least three times. Data are presented as mean ± SD. Error bars represent standard deviation (SD) for the three independent experiments. (**C**) Helical wheel projections (left panel) and three-dimensional structure simulation (right panel) of *Np*HM4. Positively charged residues are presented in red, hydrophobic residues are shown in yellow, and uncharged polar residues are shown in green. The numbers represent the positions of amino acid residues.

The MICs and MBCs of mutant AMPs (*Np*HM1–*Np*HM7) against Gram-negative and Gram-positive strains were determined (Table 3). *Np*HM7, which had the highest positive charge, appeared to have no antibacterial activity. Moreover, neither *Np*HM1 nor *Np*HM3 (mutated at one site) showed bactericidal activity against *V. alginolyticus*. For Gram-positive bacteria (*S. aureus* and *Bacillus subtilis*), none of the mutant AMPs from *Np*HM1 to *Np*HM6 had significant antibacterial activity. Eventually, it was found that only *Np*HM4 showed superior antibacterial activity against Gram-negative bacteria (*V. alginolyticus* and *V. parahaemolyticus*), with corresponding MBCs of 30 μM and 50 μM, respectively (Table 3). Furthermore, *Np*HM4 inhibited *V. alginolyticus* with MICs ranging from 5 to 25 μM (IC_50_ = 12.25 μM), and the MICs for *V. parahaemolyticus* ranged from 15 to 45 μM (IC_50_ = 32.09 μM) (Figure 2A,B). Thus, *Np*HM4 (Figure 2C) was selected for further study on its antibacterial activity and mechanism of action against Vibrios.

### 2.5. Time-Course Bactericidal Activity of NpHM4 towards V. alginolyticus and V. parahaemolyticus

**Figure 3:**
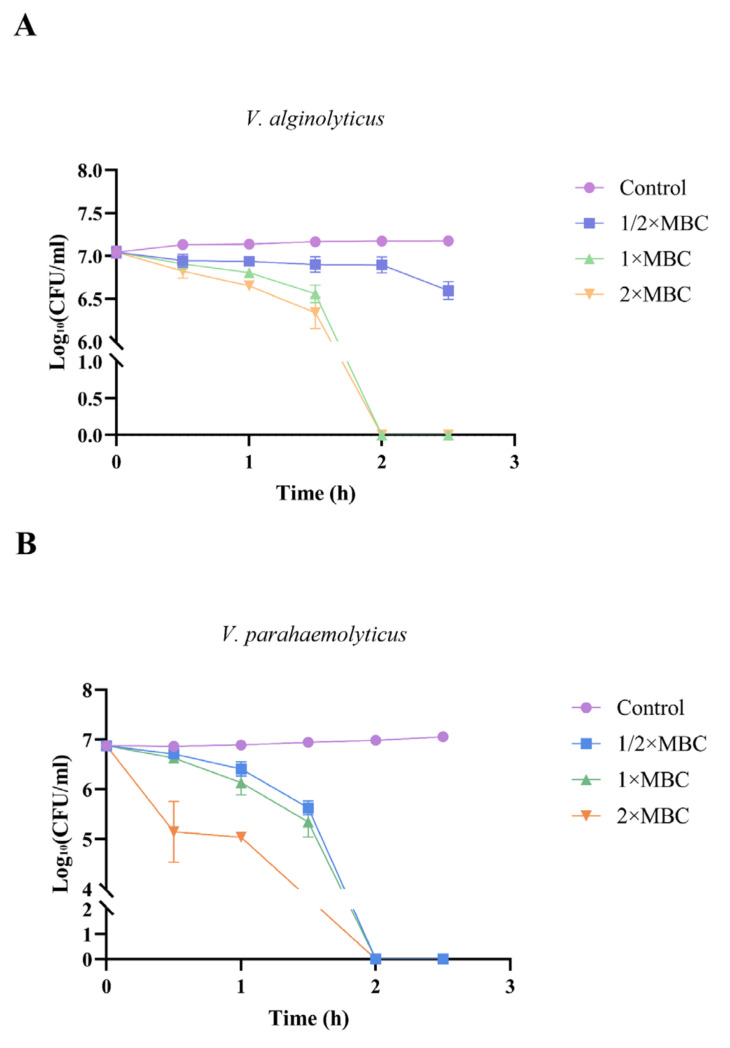
Time-killing kinetics of *Np*HM4 against *V. alginolyticus* (**A**) and *V. parahaemolyticus* (**B**) at 1/2×, 1×, and 2× MBC concentrations for 0, 0.5, 1, 1.5, 2, and 2.5 h, with PBS buffer (0.01 M, pH 7.2) as negative control.

A time-killing kinetic assay was used to evaluate the bacteria-killing dynamics of *Np*HM4, showing rapid concentration- and time-dependent bactericidal activity (Figure 3). Within 1 h, the concentration of *V. alginolyticus* was reduced from 1.1 × 10^7^ CFU/mL to approximately 6.4 × 10^6^ CFU/mL at 1× MBC of *Np*HM4. Moreover, exposure to the synthetic peptide *Np*HM4 (1× MBC) for 120 min was sufficient to obtain complete inhibition of *V. alginolyticus*, which could also be achieved with incubation at 2× MBC. After 1 h of treatment, *Np*HM4 was able to eradicate most *V. parahaemolyticus* at 1× MBC. Furthermore, *Np*HM4 completely killed *V. parahaemolyticus* within 2 h at 1× MBC and 2× MBC (no regrowth). Overall, these results confirmed that *Np*HM4 had a strong inhibitory effect on *V. alginolyticus* and *V. parahaemolyticus.*

### 2.6. Membrane-Penetrating Activity of NpHM4 toward V. alginolyticus

**Figure 4:**
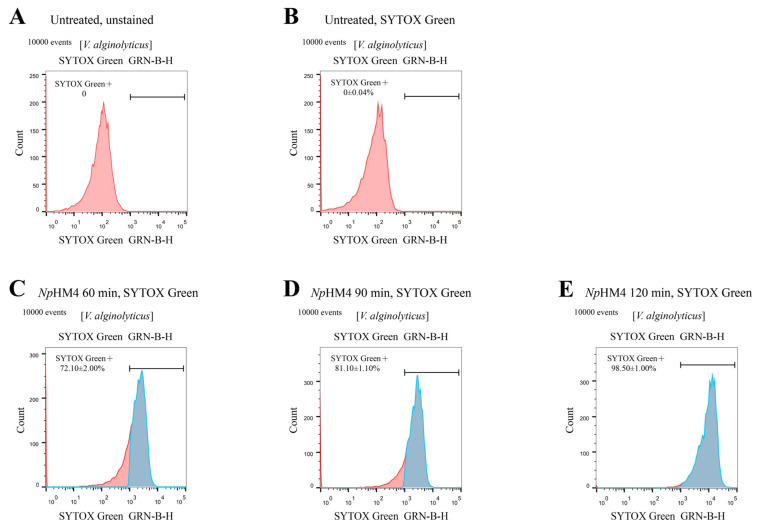
Flow cytometry analysis of *V. alginolyticus* (A056) treated with *Np*HM4. (**A**) Untreated *V. alginolyticus* without staining. (**B**) Untreated *V. alginolyticus* stained with SYTOX^®^ Green. The effect of *Np*HM4 at 2× MBC for 60 min (**C**), 90 min (**D**), and 120 min (**E**) on membrane permeability (SYTOX^®^ Green) of *V. alginolyticus*. Collected healthy cells are presented in red, and dead cells following membrane disruption in experimental group are shown in blue. The percentage of dead cell populations are shown in the center of each plot. The % values represent the shift on the *x*-axis in (**C**–**E**) (*Np*HM4 treated), compared with (**A**) (the defined fluorescence threshold in untreated cells).

The interaction of *Np*HM4 with the membrane of *V. alginolyticus* (A056) was investigated using flow cytometry. SYTOX^®^ Green will not cross intact membranes, but will fluorescently stain nucleic acids in dead cells following membrane disruption. In the absence of *Np*HM4, there was nearly no fluorescent signal (Figure 4A,B), indicating that bacterial cells do not fluoresce spontaneously. The flow cytometry results indicated that *Np*HM4-treated *V. alginolyticus* cells exhibited green fluorescence with 72.10 ± 2.00%, 81.10 ± 1.10%, and 98.50 ± 1.00% at 60, 90, and 120 min, respectively (Figure 4C–E). These results suggested that *Np*HM4 exhibited binding and penetrating activities against *V. alginolyticus* in a time-dependent manner.

### 2.7. Effect of Peptide NpHM4 on V. alginolyticus Ultrastructure via Scanning Electron Microscopy

**Figure 5:**
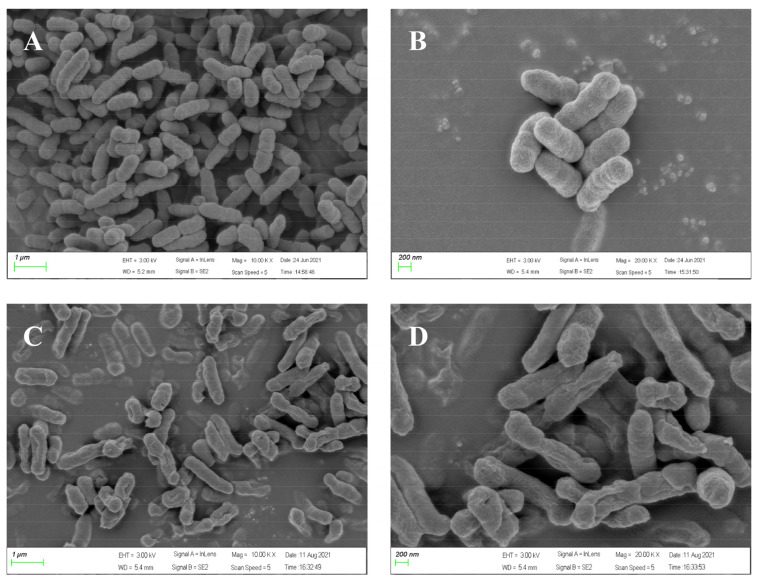
Scanning electron microscopic micrographs of *V. alginolyticus* (A056) treated with *Np*HM4. (**A**,**B**) Treated with 0.01 M, pH 7.2, PBS (control); (**C**,**D**) Treated with 1× MBC *Np*HM4 for 2 h.

To directly observe cell morphologic changes after peptide treatments, scanning electron microscopy was conducted. As shown in Figure 5A,B, bright and smooth surfaces were observed on the untreated *V. alginolyticus* cells (controls). However, *V. alginolyticus* cells treated with *Np*HM4 for 2 h showed significant membrane roughening, corrugation, and damage (Figure 5C,D).

### 2.8. Cytotoxicity of NpHM4 to HEK293 Cells

**Figure 6:**
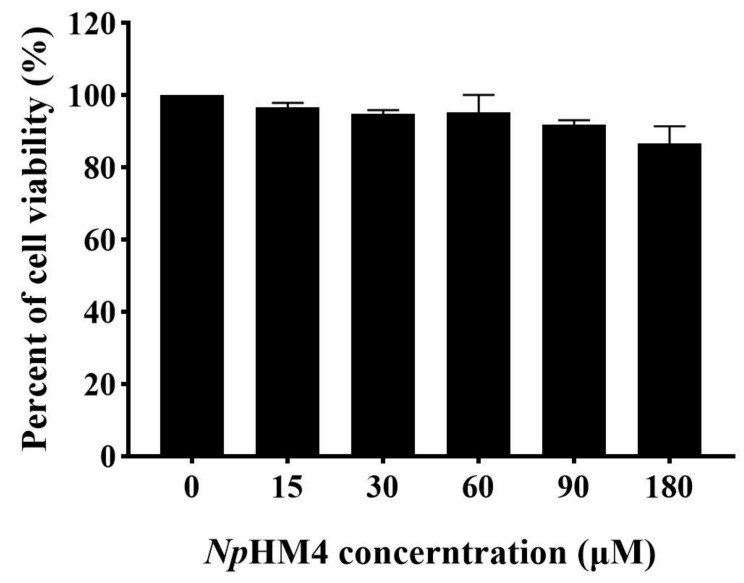
Percentages of cell viability of HEK293 cells treated with *Np*HM4 at different concentrations (0, 15, 30, 60, 90, and 180 μM). The experiments were performed in triplicate and the data were expressed as the mean ± SD. The statistical analysis was performed using one-way ANOVA and Tukey’s test.

To further explore the potential safety of *Np*HM4 for therapeutic application, its cytotoxicity was tested with human embryonic kidney 293 (HEK293) cells with the Cell Counting Kit-8 (CCK-8) assay. The diluted *Np*HM4 peptide was incubated with HEK293, and PBS-incubated cells were used as a control. No morphological changes in the cells were observed when treated with *Np*HM4 (Figure S2B–D). Under the condition of 1×MBC (30 μM), the cell survival rate could reach 94.84%, as shown in Figure 6. Moreover, at the maximum test concentration (180 μM), there was no significant difference in cell metabolic activity between the *Np*HM4-treated samples and the control (*p* > 0.05), with 86.69% of the cells surviving (Figure 6).

### 2.9. Influence on the Cell Cycle Progression

**Figure 7:**
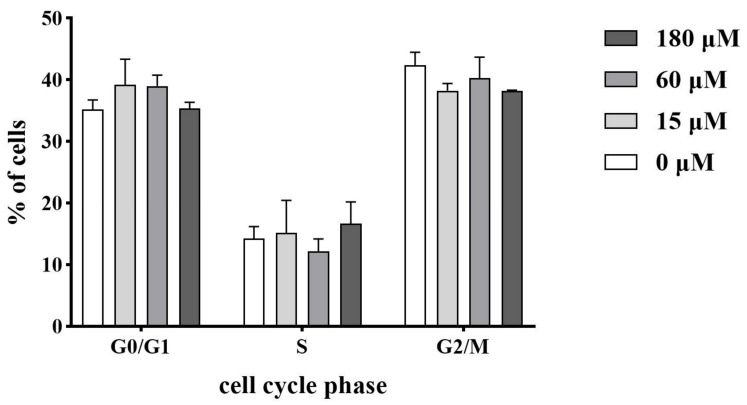
Effects of peptide *Np*HM4 on cell cycle distribution. HEK293 cells after 48 h of treatment with 0, 15, 60, and 180 μM of *Np*HM4 were collected, washed, fixed, strained, and measured using flow cytometry (Guava^®^ easyCyte, Millipore). Values are presented as mean ± SD (N = 3).

The cell cycle distribution of HEK293 cells incubated with *Np*HM4 is shown in Figure 7. It was determined that *Np*HM4 treatment caused no differences when compared with the control group (0 μM) with respect to the cell populations in the different phases of the cell cycle. In the case of the treatment with 180 μM of *Np*HM4, the percentages of the cells in the G2/M phase represented 38.2 ± 0.15%, which was not significantly different from the percentages of 42.3% ± 2.15% in the control group (*p* > 0.05).

## 3. Discussion

The emergence of pathogenic organisms with resistance to conventional antibiotics has posed a severe hazard to public health. This peril urgently necessitates the development of novel antimicrobial molecules [30]. AMPs represent a hopeful class of novel antibacterial pharmaceuticals, as they possess potent antibacterial activity [31] and the low possibility of inducing bacterial resistance [32]. In recent years, hemocyanin has been found to be involved in many immune-related activities in addition to its role as a respiratory protein [33,34,35], including the production of various AMPs constitutively or under the action of pathogens [36]. In this study, we first characterized six putative AMP sequences (*Np*HN1 to *Np*HN6), predicted from an *N. pompilius* hemocyanin sequence. These peptides featured typical AMP properties, such as high proportions of hydrophobic amino acids (≥30%) and positive charges. When determining the antibacterial activity of these six natural peptides, only the *Np*HN5 peptide showed antibacterial activity against the tested bacteria. However, *Np*HN5 did not show very powerful or broad antimicrobial activity. It has been reported that increasing the proportion of positively charged amino acids in AMPs improved the electrostatic contact between the molecule and the membrane, making it easier for the molecule to aggregate and reach the rupture threshold concentration, thereby improving antibacterial activity [37]. At the same time, a variety of structural parameters, such as amphipathicity, net charge, and hydrophobicity, have been reported to severely affect the antimicrobial effectiveness of peptides [38]. Thus, alteration of peptide sequences has been performed to improve the antibacterial activity of AMPs [39].

We modified *Np*HN5 by substituting selected hydrophobic amino acids with arginine (R), which resulted in an increased net positive charge. We found that *Np*HM7 had no inhibitory activity, despite having the highest positive charge. This suggests that increasing the positive charge content alone does not increase the antibacterial activity. It has been stated that high core hydrophobicity can lead to an increased potential to peptide self-association at the membrane surface (and possibly precipitation), thereby limiting the concentration of peptides that actually affect the bacterial membrane and consequently reducing the inhibitory activity [40,41]. Among *Np*HM1–*Np*HM3, *Np*HM1 and *Np*HM3 showed similar inhibitory effects against *E. coli* and *V. parahaemolyticus*, whereas *Np*HM2 showed slightly lower inhibitory activity against Gram-negative bacteria than *Np*HM1 and *Np*HM3, a phenomenon which may be attributed to the fact that Arg does not disrupt its core hydrophobic segment. Compared with *Np*HM5 and *Np*HM6, *Np*HM4, with an α-helix conformation, possessed a suitable positive charge and amphipathicity, which significantly increased its antibacterial activity against *V. alginolyticus* and *V. parahaemolyticus*. These abovementioned results suggest that charge distribution itself does not dominate the impact on activity, but rather requires a good balance among amphiphilicity, positive charge, and hydrophobicity to effectively enhance the activity of the antimicrobial peptide, which was consistent with previous studies [40,42].

The antibacterial mechanism of cationic α-helical AMPs normally begins with their binding to bacteria via electrostatic attraction. Then, hydrophobic forces propel the peptide to be inserted into the cytoplasmic membrane, resulting in the disruption of the membrane [43,44]. Other bactericidal mechanisms of AMPs can also occur in several disparate ways, such as the formation of barrel wall holes or annular holes and their aggregation on the cell surface [45]. Here, SYTOX^®^ Green was applied to determine the membrane permeability of *V. alginolyticus* post incubation with *Np*HM4, which showed dose-dependent and time-dependent penetration activity. The SEM results further confirmed that the killing mechanism of *Np*HM4 was cell membrane rupture, leading to intracellular content infiltration and cell death. Similar results were recently reported, in which a novel hemoglobin-derived β-sheet peptide from *Penaeus vannamei, Pv*HS9, showed antimicrobial activity against *V. parahaemolyticus* and *S. aureus* due to membrane penetration [46]. Similarly, Chensinin-1b, a peptide derived from skin secretions of *Rana chensinensis*, was shown to attenuate the growth of *E. coli* through electrostatic interactions with *E. coli* anionic cell membranes [47]. The detailed role of *Np*HM4 in bacterial membranes is unclear and needs further investigation.

The cytotoxicity of AMPs is another important feature associated with the development of antimicrobial drugs, as it limits their security and application. Here, we confirmed that *Np*HM4 had low impact on mammalian cell viability. In total, 81.29% of L929 cells were viable when treated at the maximum test concentration (250 µg/mL) with the novel PA-13 peptide designed by Natthaporn et al. [25]. Similarly, the *Pv*H4a peptide, derived from histone H4 of *Penaeus vannamei*, had no adverse effects on normal human hepatocytes (LO2 cells) even at high concentrations (249.6 µg/mL), with a cell survival rate of more than 90.3% [48]. In the present study, we also confirmed that *Np*HM4 does not disrupt the culture of HEK293 cells. This is consistent with the results of Kim et al., who demonstrated that amoxicillin does not affect the cell cycle of HEK293 [49]. In contrast, cytotoxic mycotoxins induced G1 cell cycle arrest and apoptosis in the cells [50]. Thus, it can be concluded that novel AMPs derived from animals often show low cytotoxicity to animal cells, suggesting a huge and promising library for AMPs’ development.

## 4. Materials and Methods

### 4.1. Prediction of AMPs from N. pompilius Hemocyanin

Two online software were applied to develop AMPs from the *N. pompilius* hemocyanin. The *N. pompilius* hemocyanin was obtained from our database, which has been published [22]. First, the antimicrobial peptide server (AntiBP: https://webs.iiitd.edu.in/raghava/antibp/index.html/; accessed on 19 April 2021) was utilized to search for potential AMPs in the *N.* *pompilius* hemocyanin sequence [51]. Then, the predicted AMPs were validated using the predict antimicrobial region within peptides tool from CAMP (http://www.camp.bicnirrh.res.in/; accessed on 19 April 2021) [52]. The peptide length was set to 15–16, and one algorithm with high accuracy, the support vector machine (SVM), was selected for use in the prediction [53]. The SVM algorithm provided AMP probability scores. The antimicrobial peptide calculator and predictor (APD3: https://wangapd3.com/prediction/prediction_main.php/; accessed on 19 April 2021) was then utilized to determine the physicochemical properties and the structure of the predicted peptides (N1–N44 and *Np*HN1–*Np*HN6) [54].

### 4.2. Mutant Peptide Design on the Basis of NpHN5

The positive charge of an AMP depends on the amount of arginine (Arg), lysine (Lys), and histidine (His) in the sequence, among which, Arg can form strong bidentate hydrogen bonds with the phosphate portion of both lipid head groups, thereby promoting deeper insertion into the membrane and giving the AMP a greater capacity for membrane disruption [55]. Similarly, hydrophobicity and amphiphilicity are also key structural and physicochemical parameters for the design of novel antimicrobial peptides. According to these parameters, seven different mutant peptides (*Np*HM1–*Np*HM7) were designed from the *Np*HN5 peptide sequence. First, we modified *Np*HN5 by simultaneously replacing three different sites of hydrophobic amino acids (Phe^3^, Leu^8^, and Ala^15^) with arginines to obtain the *Np*HM7 peptide. In designing this peptide, Phe (position 3) and Leu (position 8) were chosen for substitution due to their strong hydrophobicity [56] and their frequent occurrence in membrane domains, whereas the positively charged Arg residues replaced Ala (position 15) to maintain cationic properties and enhance peptide solubility [40]. Then, we synthesized *Np*HM1–*Np*HM3 and *Np*HM4–*Np*HM6, on the one hand, to compare the antibacterial activity of peptides with different positive charge ratios, and on the other hand, to investigate whether different positive charge distributions affect the activity of mutant peptides with the same hydrophobicity level. *Np*HM1, *Np*HM2, and *Np*HM3 were designed via the alteration of three hydrophobic amino acids into arginine (Leu^8^, Ala^15^, and Phe^3^). *Np*HM4 was obtained with the substitution of two hydrophobic amino acids (Leu^8^ and Ala^15^). The *Np*HM5 and *Np*HM6 peptides were modified in a similar manner, with simultaneous amino acid substitutions with Arg at positions Phe^3^ and Ala^15^ and Phe^3^ and Leu^8^, respectively. Three sites (Phe^3^, Leu^8^, and Ala^15^) were simultaneously replaced by three arginines to obtain the *Np*HM7 peptide.

The 3D structures of the mutant peptides (*Np*HM1–*Np*HM7) were initially predicted with the I-TASSER online server (https://zhanglab.ccmb.med.umich.edu/I-TASSER/; accessed on 21 May 2021) [57] and edited on the PyMol program. The helical wheel projection was made with the online program NetWheels: Peptides Helical Wheel and Net projections maker (http://lbqp.unb.br/NetWheels/; accessed on 23 May 2021) [58]. The mean hydrophobic moment obtained from Heliquest online program (http://heliquest.ipmc.cnrs.fr/; accessed on 6 June 2021) [18].

### 4.3. Peptide Synthesis

Six predicted AMPs (*Np*HN1, *Np*HN2, *Np*HN3, *Np*HN4, *Np*HN5, and *Np*HN6) derived from the hemocyanin of *N. pompilius* and seven mutants from *Np*HN5 (*Np*HM1, *Np*HM2, *Np*HM3, *Np*HM4, *Np*HM5, *Np*HM6, and *Np*HM7) were synthesized by Guopeptide Biotechnology (Hefei, China), with a purity of more than 95%. The molecular mass of the synthetic peptides was ascertained using liquid chromatography coupled with mass spectrometry (LC-MS/ESI). All the processes were carried out according to the company’s standard protocols.

### 4.4. Antimicrobial Assay

The antimicrobial activity of the peptides was determined against Gram-negative bacteria, including *V. alginolyticus* (A056), *E. coli* (DH5α), and *V. parahaemolyticus* (2013V-1174), which were obtained from the South China Sea Institute of Oceanology, Chinese Academy of Sciences. The activity was also tested against the Gram-positive bacteria *S. aureus* ATCC 25923 and *B. subtilis* ATCC 6051. To assess the antimicrobial activities of the peptides, bacterial cells were cultured to the logarithmic growth phase and then diluted to 10^6^–1.5 × 10^7^ CFU/mL with PBS (0.01 M, pH 7.2). Equal volumes of serially diluted peptides, with concentrations ranging from 30 to 450 μM in PBS, and bacteria suspension were mixed and incubated at room temperature for 2 h, respectively. Then, 6 μL of each serially diluted mixture was plated on sterile 12-well agar plates, followed by incubation for 11 to 12 h, and the colonies were assessed to determine the MICs and MBCs. The MICs were expressed (in µM) as an [a]–[b] concentration interval, where [a] is the highest peptide concentration at which bacteria were growing and [b] is the lowest concentration that caused 100% growth inhibition. Cultures without peptides were employed as controls. All the tests were performed in triplicate and repeated three times.

### 4.5. Time-Course Bactericidal Activity of NpHM4

The bacterial-killing kinetics of *Np*HM4 were assessed by evaluating the time-course bactericidal activity of *V. alginolyticus* and *V. parahaemolyticus*, as described previously [59]. Bacteria suspensions (10^6^–1.5 × 10^7^ CFU/mL) were incubated with *Np*HM4 at certain concentrations (1/2×, 1×, and 2× MBC) at 30 °C. Then, 6 μL of each serially diluted mixture was plated on an agar plate at different time intervals (0, 0.5, 1, 1.5, 2, and 2.5 h). Bacteria were incubated at 30 °C for 11 to 12 h to determine the colonies. Cultures without peptides were employed as controls. All the tests were performed in triplicate and repeated three times.

### 4.6. Membrane Permeability

To assess the membrane permeability induced by *Np*HM4, the high-affinity nucleic acid stain SYTOX^®^ Green (S7020, Thermo Fisher, Waltham, MA, USA) was used, as it easily penetrates cells with compromised plasma membranes [60]. Briefly, *V. alginolyticus* was cultured to the logarithmic growth phase and harvested via centrifugation at 2000× *g* for 5 min. The bacterial cells were washed twice with PBS and resuspended to 10^7^ CFU/mL in PBS. Then, the bacterial suspensions were treated with *Np*HM4 at concentrations of 2× MBC and incubated for 60, 90, or 120 min at 30 °C. SYTOX^®^ Green at a final concentration of 1 μM was subsequently added to each sample. Untreated bacterial cells (without peptides) served as negative controls. The cells with no SYTOX^®^ Green and no peptides were set as a blank control. The cells were then subjected to flow cytometry (Guava^®^ easyCyte, Millipore, Billerica, MA, USA) so that the extent of membrane permeabilization could be assessed. The experiments were performed in triplicate and analyzed using FlowJo software. The permeabilization efficiency was determined by the percentage of the cells’ fluorescence.

### 4.7. Analysis of Cellular Morphology via Scanning Electron Microscopy (SEM)

The morphological changes in bacteria after treatment with *Np*HM4 were observed using scanning electron microscopy (SEM). As previously described [58], *V. alginolyticus* was cultured in thiosulphate-citrate-bile salt sucrose (TCBS) agar to the logarithmic growth phase and harvested via centrifugation at 2000× *g* for 5 min. Cell pellets were washed twice with PBS and resuspended. Then, the cell suspension was incubated at 30 °C for 120 min with *Np*HM4 at 1× MBC. Subsequently, the cells were centrifuged at 8000 rpm/min for 15 min and washed twice with PBS. Bacterial pellets were then fixed in 2.5% (vol/vol) glutaraldehyde at 4 °C overnight, followed by washing twice in PBS and dehydration in a graded ethanol series (50%, 70%, 90%, and 100%) for 15 min, respectively. Finally, all the samples were transferred to ethanol–tertiary butanol mixtures (1:1) and then transferred to pure tertiary butanol for a 20 min incubation. The specimens were observed after lyophilization and gold-coating, using a scanning electron microscope (Zeiss Sigma 300, Jena, Germany).

### 4.8. Cytotoxicity Assay

The HEK293 cells were purchased from the Shanghai Cell Bank, Chinese Academy of Sciences. Dulbecco’s modified Eagle’s medium (DMEM) containing 10% fetal bovine serum (FBS) was used to culture the HEK293 cells. All the cells were cultured at 37 °C in a carbon dioxide incubator overnight. The DMEM and FBS were purchased from Thermo Fisher Scientific (11995065, Grand Island, NY, USA; 10099141C, New Zealand, Australia).

The cytotoxicity of *Np*HM4 to HEK293 cells was assessed using the Cell Counting Kit-8 (CCK-8, K1018, APE×BIO, Technology LLC, Houston, TX, USA). As previously described [61], 6 × 10^4^ cells were seeded into the wells of a 96-well microtiter plate and incubated at 37 °C for 48 h. Then, the supernatant medium was removed from each well, and 100 μL graded concentrations of DMEM containing *Np*HM4 were added (15, 30, 60, 90, and 180 μM, n = 3 for each concentration). The HEK293 cells cultured with DMEM (0 μM *Np*HM4) constituted the control group. After incubation at 37 °C for 24 h, a CCK-8 solution (10 μL per well) was added, and the cell metabolism was quantified after incubation for 2 h at 37 °C. The absorbance was measured at 450 nm. The cell viability reflected the cytotoxicity of *Np*HM4 to cells, and the viability was calculated as follows:

% Viability = (OD_Treated_ − OD_Blank_)/(OD_Untreated_ − OD_Blank_) × 100

### 4.9. Cell Cycle Analysis via Flow Cytometry

The cell cycle analysis was tested using the cell cycle and apoptosis analysis kit (BL114A, Biosharp Biotechnology, Hefei, China). As previously described [62], the HEK293 cells were seeded at a density of 400,000 cells/well into 6-well plates (Corning Costar Corporation, Corning, NY, USA). After incubation at 37 °C in 5% CO_2_ for 24 h, the growth medium was replaced with fresh medium containing 15, 60, and 180 μM *Np*HM4 and incubated for 48 h. The HEK293 cells cultured with DMEM (0 μM *Np*HM4) constituted the control group. After the exposure, the cells were harvested via trypsinization. Single-cell suspensions were centrifuged at 1000 rpm for 5 min, washed twice with ice-cold PBS, and fixed in 70% ethanol for at least 2 h at 4 °C. The cells were rinsed again with PBS and stained with 0.5 mL of propidium iodide/RNase A staining buffer for 30 min at 37 °C, according to the manufacturer’s recommendations. Flow cytometric analysis was carried out on flow cytometry (Guava^®^ easyCyte, Millipore, Billerica, MA, USA). Twenty thousand events were obtained for each sample, and the percentages of cells in the G0/G1, S, and G2/M phases of the cell cycle were determined using FlowJo v10 software (BD Life Sciences, Louisville, KY, USA).

### 4.10. Statistical Analysis

All the tests were performed in triplicate and repeated three time. Statistical analysis was performed with a one-way analysis of variance and Tukey’s test. Significant differences were defined as *p* < 0.05.

## 5. Conclusions

In conclusion, a peptide (*Np*HN5) derived from the hemocyanin of *N.* *pompilius* was modified through the alteration of hydrophobic amino acid into arginine, obtaining seven mutant peptides (*Np*HM1–*Np*HM7). Antibacterial analysis revealed that the mutant *Np*HM4 peptide showed superior antibacterial activity against *Vibrio* spp., with the physicochemical characteristics of typical antimicrobial peptides, which contributed to the destruction of the integrity of bacterial cell membranes to exert their antibacterial activity. Moreover, *Np*HM4 had low cytotoxicity to mammalian cells, suggesting its therapeutic potential. Our study supports the validity of the prediction and optimization of novel antimicrobial agents and provides a novel AMP from ancient cephalopods with potential applications.

## Figures and Tables

**Table 1:** Antibacterial effects of the six potential antimicrobial peptides.

Peptides	Gram-Negative Bacteria			
	*Escherichia coli* (DH5α)		*Vibrio alginolyticus* (A056)	
	MIC (μM)	MBC (μM)	MIC (μM)	MBC (μM)
*Np*HN1	250–450	>450	50–260	>260
*Np*HN2	200–500	>500	100–280	>280
*Np*HN3	250–450	>450	50–250	>250
*Np*HN4	200–600	>600	100–300	>300
*Np*HN5	200–450	450	50–250	250
*Np*HN6	250–500	>500	100–300	300

Note: MIC, minimal inhibitory concentration; MBC, minimal bactericidal concentration.

**Table 2:** Mutants based on peptide *Np*HN5.

No.	Peptide Sequence	Pho% ^c^	Net Charge	Number of Mutations	Measured MW ^a^	Secondary Structure	uH ^b^
*Np*HM1	RVFAGFLRHGIKRSA	46	4.25	1	1715.02	α-helix	0.423
*Np*HM2	RVFAGFLLHGIKRSR	46	4.25	1	1757.10	β-chain	0.336
*Np*HM3	RVRAGFLLHGIKRSA	46	4.25	1	1681.00	β-chain	0.181
*Np*HM4	RVFAGFLRHGIKRSR	40	5.25	2	1800.13	α-helix	0.467
*Np*HM5	RVRAGFLLHGIKRSR	40	5.25	2	1766.11	β-chain	0.160
*Np*HM6	RVRAGFLRHGIKRSA	40	5.25	2	1724.03	α-helix	0.237
*Np*HM7	RVRAGFLRHGIKRSR	33	6.25	3	1809.14	α-helix	0.281

^a^ MW, molecular weight (g/mol) measured via mass spectroscopy (MS). ^b^ μH, the mean hydrophobic moment. ^c^ Pho%, the percentage of hydrophobic residues.

**Table 3:** MICs and MBCs of seven mutant peptides derivatives against five strains of Gram-positive and Gram-negative bacteria.

Peptides	Gram Negative						Gram Positive			
	*E. coli* DH5α		*V. alginolyticus* A056		*Vibrio parahaemolyticus* 2013V-1174		*Staphylococcus aureus* ATCC 25923		*Bacillus subtilis* ATCC 6051	
	MIC (μM)	MBC (μM)	MIC (μM)	MBC (μM)	MIC (μM)	MBC (μM)	MIC (μM)	MBC (μM)	MIC (μM)	MBC (μM)
*Np*HM1	15–60	>60	50–150	>150	15–60	>60	>60	>60	>60	>60
*Np*HM2	30–60	>50	70–150	>75	30–60	>60	>50	>50	>50	>50
*Np*HM3	15–50	>60	30–75	>150	15–60	>60	>60	>60	>60	>60
*Np*HM4	50–150	>150	5–25	30	15–45	50	>50	>50	>50	>50
*Np*HM5	>50	>50	15–70	>70	15–70	>70	>50	>50	>50	>50
*Np*HM6	>50	>50	>70	>70	15–60	>60	>50	>50	>50	>50
*Np*HM7	>150	>150	>150	>150	>150	>150	>150	>150	>150	>150

## Data Availability

Not applicable.

## Supplementary Materials

The following supporting information can be downloaded at: https://www.mdpi.com/article/10.3390/md20070459/s1, Table S1: Prediction of antimicrobial peptides derived from hemocyanin of N. pompilius; Table S2: Predictive antimicrobial peptides synthesized after screening; Figure S1: Helical wheel projections of NpHM1, NpHM4, NpHM6, and NpHM7; Figure S2: Cytotoxicity of NpHM4 against HEK293 cells.
