# Supplementary material for: Functional Characterization, Antimicrobial Effects, and Potential Antibacterial Mechanisms of NpHM4, a Derived Peptide of Nautilus pompilius Hemocyanin
Source: Mar Drugs. 2022 Jul 16;20(7):459. doi: 10.3390/md20070459 (PMC9317327; doi:10.3390/md20070459)
Supplement: Supplementary file 1 [file marinedrugs-20-00459-s001.zip › marinedrugs-1725412-supplementary.pdf]

Supplementary Information

Functional characterization, antimicrobial effects, and potential antibacterial mechanisms of *Np*HM4, a derived peptide of *Nautilus pompilius* hemocyanin

Chun Yuan [1,2], Xiaoying Zheng [1,3], Kunna Liu [4,5], Wenbin Yuan [1,3], Yang Zhang [4,5], Fan Mao [4,5,*] and Yongbo Bao [1,2,*]

[1] Zhejiang key Laboratory of Aquatic Germplasm Resources, College of Biological and Environmental Sciences, ZhejiangWanli University, Ningbo 315100, China; 2020881038@zwu.edu.cn (C.Y.); xyzheng20@163.com (X.Z.); yuanwenbin2021@163.com (W.Y.)
[2] Ninghai Institute of Mariculture Breeding and Seed Industry, Zhejiang Wanli University, Ninghai 315604, China
[3] School of Marine Science, Ningbo University, Ningbo 315211, China;
[4] CAS Key Laboratory of Tropical Marine Bio-Resources and Ecology and Guangdong Provincial Key Laboratory of Applied Marine Biology, South China Sea Institute of Oceanology, Innovation Academy of South China Sea Ecology and Environmental Engineering, Chinese Academy of Sciences, Guangzhou 510301, China; liukunna18@mails.ucas.ac.cn (K.L.); yzhang@scsio.ac.cn (Y.Z.)
[5] Southern Marine Science and Engineering Guangdong Laboratory (Guangzhou), Guangzhou 510301, China
* Correspondence: maofan@scsio.ac.cn (F.M.); baoyongbo@zwu.edu.cn (Y.B.); Tel.: +86-20-8910-2507 (F.M.)

Table S1. Prediction of antimicrobial peptides derived from hemocyanin of *N. pompilius*.

No.	Peptide sequence	Pho%[c]	Net charge	Score	MW[a]	Secondary structure
N1	VFAGFMLHGFKKSAL	60	2.25	0.975	1653.02	α-helix
N2	FAGFMLHGFKKSALV	60	2.25	0.967	1653.02	α-helix
N3	GFKKSALVSFSIVHH	46	2.5	0.965	1656.94	α-helix
N4	LHGFKKSALVSFSIV	53	2.25	0.948	1632.96	α-helix
N5	KALHRYACCIHGMAT	53	2.5	0.891	1675.02	β-chain
N6	FKKSALVSFSIVHHQ	46	2.5	0.882	1728.02	α-helix
N7	GFLLHGIQASANVRI	53	1.25	0.882	1595.86	α-helix
N8	RVFAGFMLHGFKKSA	53	3.25	0.875	1696.05	α-helix
N9	RVFAGFLLHGIKRSA	53	3.25	0.874	1671.99	α-helix
N10	FRLSGIHTSANVKVL	46	2.25	0.85	1641.93	α-helix
N11	IFVPAHVKSMNISHK	46	2.5	0.839	1708.06	α-helix
N12	IIPAPNIIFVPAHVK	60	1.25	0.835	1629.02	-
N13	FVPAHVKSMNISHKG	40	2.5	0.821	1651.95	-
N14	RIFAGFLLHGIGQSA	53	1.25	0.804	1586.85	α-helix
N15	RISGGPIIRKNINKL	33	4	0.802	1679.04	-
N16	ANVRIYICVPTQRGS	40	2	0.784	1676.96	-
N17	RIWAIWQALQIYRGK	53	3	0.781	1902.28	α-helix
N18	FLFWGIHTSANVKVF	60	1.25	0.778	1766.07	α-helix
N19	QCKPYACCQHGMPTF	40	1.25	0.769	1714.05	-
N20	GFLFWGIHTSANVKV	53	1.25	0.764	1675.95	α-helix
N21	FASFRLSGIHTSANVKVL	46	1.25	0.752	1947.27	α-helix
N22	QIDRKNLVVRKDLRR	33	4	0.746	1909.27	-
N23	NRVFAGFMLHGFKKS	46	3.25	0.726	1739.07	α-helix
N24	MLHGFKKSALVSFSI	53	2.25	0.702	1665.03	α-helix
N25	VFAGFLLHGIKRSAD	53	1.25	0.669	1630.91	α-helix
N26	PLCPNPKALHRYACC	46	2.25	0.659	1686.06	-
N27	FAGFLLHGIKRSADV	53	1.25	0.656	1630.91	α-helix
N28	GLPLQCKPYACCQHG	40	1.25	0.649	1617.94	-
N29	AIWQALQIYRGKHYL	46	2.25	0.646	1860.19	α-helix
N30	AIAAYHGLPLQCKPY	46	1.25	0.623	1644.96	-
N31	IIWQELQKIRGLSGH	40	1.25	0.622	1778.09	α-helix
N32	YHGLPLQCKPYACCQ	40	1.25	0.601	1724.06	-
N33	IWQALQIYRGKHYLA	46	2.25	0.598	1860.19	α-helix
N34	EINRRRSRNRVFAGF	33	4	0.583	1878.14	-
N35	GFLLHGIKRSADVNF	46	1.25	0.581	1673.94	α-helix
N36	LQCKPYACCQHGMPT	40	1	0.579	1680.03	-
N37	PLQCKPYACCQHGMP	40	1.25	0.558	1676.04	-
N38	ALQIYRGKHYLAHCA	46	2.5	0.537	1744.05	α-helix

No.	Peptide sequence	Pho%[c]	Net charge	Score	MW[a]	Secondary structure
N39	ISGGPIIRKNINKLT	33	3	0.529	1623.96	-
N40	KIHSRQSHPRVFAGF	33	3.5	0.522	1767.03	α-helix
N41	PNPKALHRYACCIHG	40	2.5	0.519	1679.99	β-chain
N42	HQIDRKNLVVRKDLR	33	3.25	0.515	1890.22	-
N43	IWQALQQYRRIPYNK	33	3	0.513	1977.30	α-helix
N44	RNRVFAGFMLHGFKK	46	4.25	0.499	1808.18	α-helix

[a] MW, molecular weight (g/mol)predicted via APD3.

[c] Pho%, the percentage of hydrophobic residues.

"-" indicates the structure of the predicted peptide was unclear.

Table S2. Predictive antimicrobial peptides synthesized after screening.

No.	Peptide sequence	Pho%[c]	Net charge	Score	Measured MW[a]	Secondary structure
*Np*HN1	VFAGFMLHGFKKSALV	62	2.25	0.975	1752.13	α-helix
*Np*HN2	FASFRLSGIHTSANVKVL	50	2.25	0.894	1947.24	α-helix
*Np*HN3	KALHRYACCIHGMAT	53	2.5	0.891	1675.02	β-chain
*Np*HN4	GFLLHGIQASANVRI	53	1.25	0.882	1595.86	α-helix
*Np*HN5	RVFAGFLLHGIKRSA	53	3.25	0.874	1671.99	α-helix
*Np*HN6	RIFAGFLLHGIGQSA	53	1.25	0.804	1586.85	α-helix

[a] MW, molecular weight (g/mol) measured via mass spectroscopy (MS).

[c] Pho%, the percentage of hydrophobic residues.

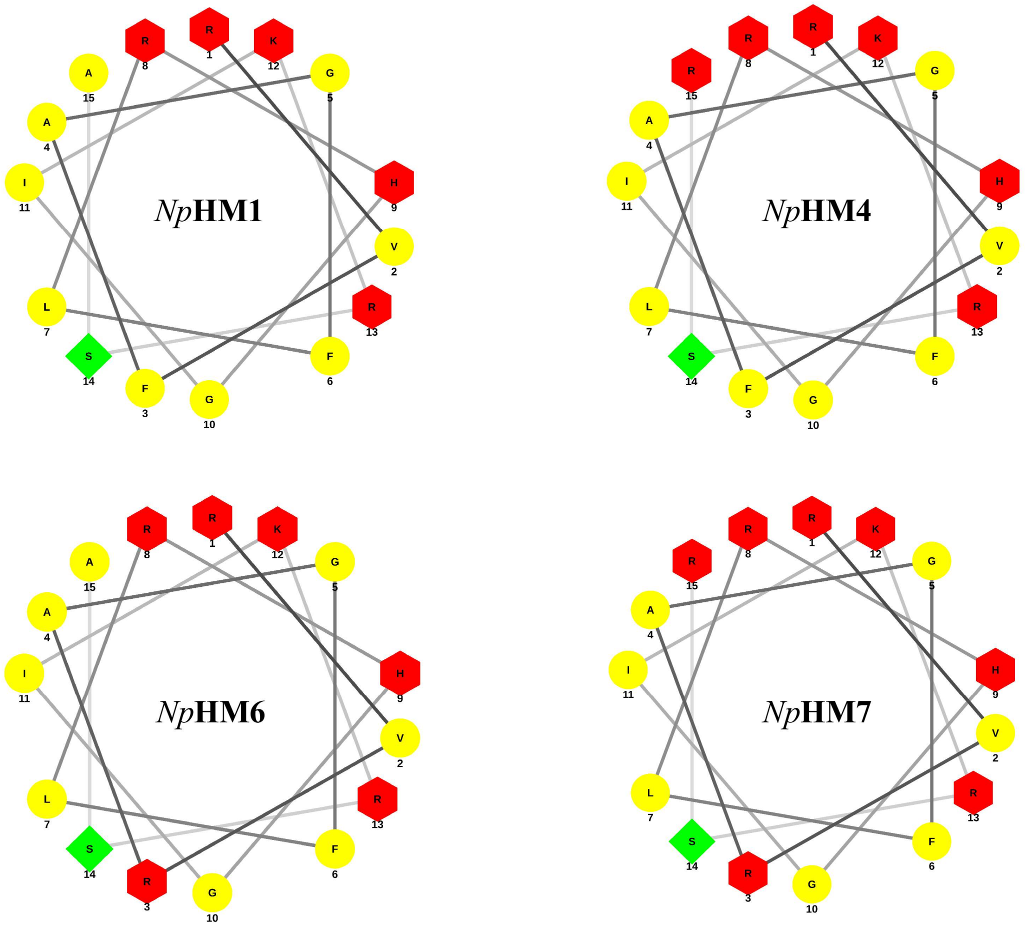

Figure S1. Helical wheel projections of *Np*HM1, *Np*HM4, *Np*HM6, and *Np*HM7. Positively charged residues are presented in red, hydrophobic residues are shown in yellow, and uncharged polar residues are shown in green. The numbers represent the positions of amino acid residues.

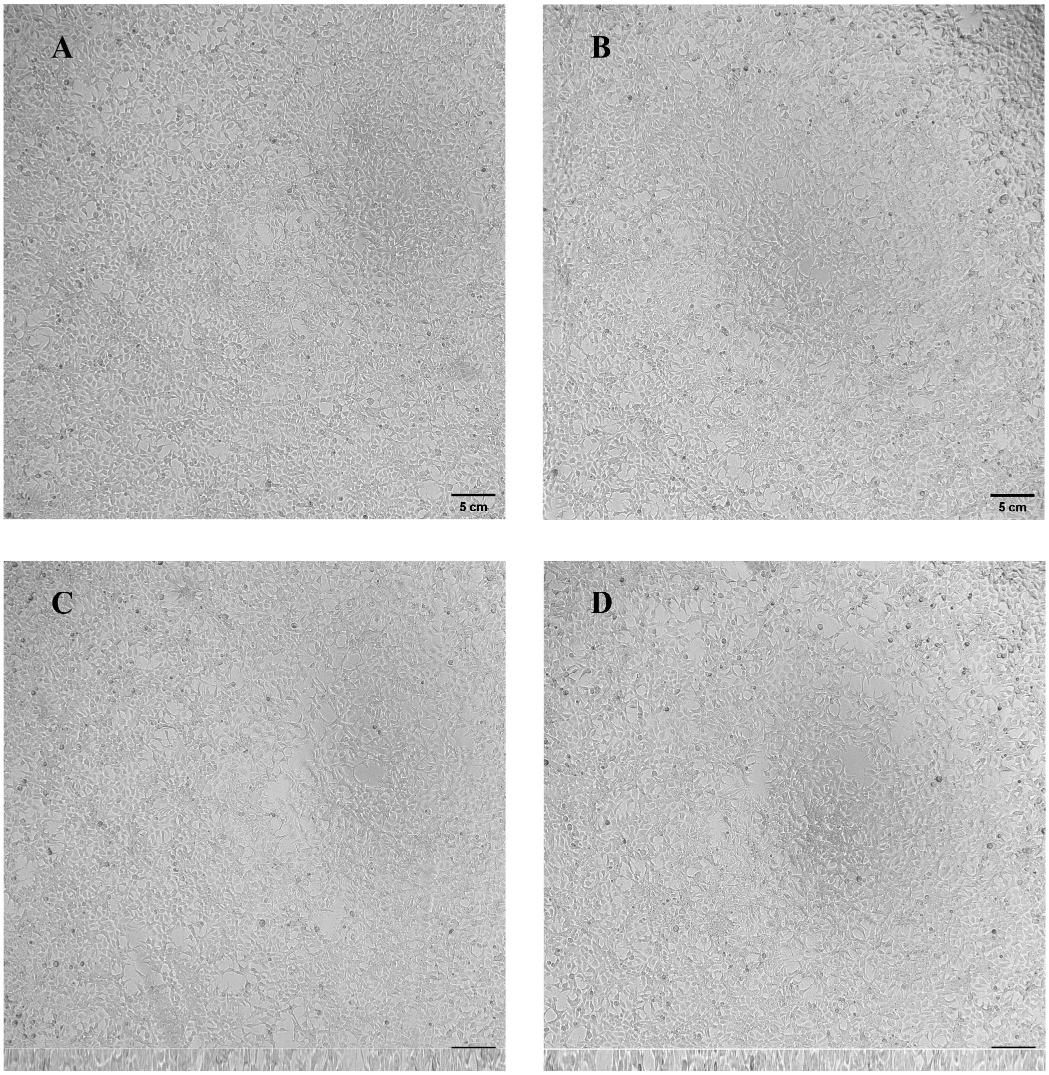

Figure S2. Cytotoxicity of *Np*HM4 against HEK293 cells. (A-D) Representative images of *Np*HM4-treated HEK293 cells at 24 h post treatment. (A) Untreated (control); (B) 30 μM *Np*HM4; (C) 90 μM *Np*HM4; (D) 180 μM *Np*HM4.
